# Hepatitis reactivation in patients with rheumatic diseases after immunosuppressive therapy—a report of long-term follow-up of serial cases and literature review

**DOI:** 10.1007/s10067-013-2450-9

**Published:** 2013-12-11

**Authors:** Dandan Xuan, Yiqi Yu, Linyun Shao, Jiali Wang, Wenhong Zhang, Hejian Zou

**Affiliations:** 1 Department of Rheumatology, Huashan Hospital affiliated to Fudan University, 12 Wulumuqi Zhong Rd, Shanghai, 200040 China; 2 Department of Infectious Diseases, Huashan Hospital affiliated to Fudan University, 12 Wulumuqi Zhong Rd, Shanghai, 200040 China; 3 Institutes of Biomedical Sciences, Fudan University, Shanghai, 200032 China; 4 MOH and MOE Key Laboratory of Medical Molecular Virology, Shanghai Medical College, Fudan University, Shanghai, 200032 China

**Keywords:** Disease-modifying anti-rheumatic drugs, Hepatitis B, Rheumatic disease, Steroid, Tumor necrosis factor-alpha-blocking agent

## Abstract

The aims of this paper are to report hepatitis B virus reactivation in 12 patients with rheumatic disease undergoing immunosuppressive therapy and to evaluate whether pre-emptive antiviral therapy is necessary in patients receiving disease-modifying anti-rheumatic drugs. From January 2008 to March 2012, a total of 12 HBV-infected patients with rheumatic diseases were consecutively enrolled in the long-term follow-up. Liver function, HBV DNA, and serum aminotransferase level were tested during the follow-up. We also reviewed the published reports and summarized the clinical characteristics of HBV reactivation during immunosuppressive therapy in patients with rheumatic diseases. The medium duration of follow-up was 41 months (range 16–48). Patients were treated with prednisone, disease-modifying anti-rheumatic drugs (DMARDs) or tumor necrosis factor-alpha-blocking agents (TNFBA). HBV reactivation was only documented in two patients treated with prednisone without pre-emptive antiviral therapy. One hundred patients from literature review were identified as having HBV reactivation; 20.8 % of the patients receiving prednisone experienced HBV reactivation compared to only 4.46 and 9.52 % of patients treated with DMARDs or TNFBA, respectively. This long-term follow-up of serial cases suggests that pre-emptive antiviral therapy should be administered in patients receiving prednisone therapy for rheumatic disease. In contrast, DMARDs and TNFBA are relatively safe to HBV-infected patients with rheumatic diseases. Close monitoring of HBV DNA and ALT levels is necessary in the management of HBV reactivation.

## Introduction

Hepatitis B virus (HBV) infection is a global health problem, resulting in more than 350 million people worldwide [1]. Chronic infection with HBV can lead to cirrhosis, hepatic decompensation, and hepatocellular carcinoma.

HBV reactivation in patients undergoing chemotherapy or immunosuppressive therapy has been a well-recognized complication [2]. However, most of these reports have come from the fields of oncology and transplantation. The emergence of immunosuppressive therapy as a key therapeutic option for patients with rheumatoid disease has been associated with increasing reports of HBV reactivation.

EASL clinical practice guidelines updated its recommendations for management of chronic hepatitis in 2012, claiming that HBsAg-positive candidates for chemotherapy and immunosuppressive therapy should be tested for HBV DNA levels and should receive pre-emptive nucleotide or nucleoside analogue administration during therapy (regardless of HBV DNA levels) and lasting for 12 months after cessation of therapy [3]. However, pre-emptive therapy in patients with rheumatic diseases treated with disease-modifying anti-rheumatic drugs (DMARDs) or tumor necrosis factor-alpha-blocking (TNFBA) is still a matter of controversy.

We conducted this long-term follow-up of serial cases and literature review to access and summarize the current evidence of HBV reactivation in HBV-infected patients with rheumatic diseases who receive different immunosuppressive therapy, including steroids, DMARDs, and TNFBA. We also evaluated whether pre-emptive antiviral therapy is necessary in different drug administration.

## Materials and methods

### Patients

From January 2008 to March 2012, HBV-infected patients who were candidates for immunosuppressive therapy for newly diagnosed rheumatic diseases were consecutively enrolled in the long-term follow-up. Patients were excluded if they had the evidence of autoimmune hepatitis, prior exposure to immunosuppressive therapy or coinfection with hepatitis C or D before the administration. Finally, a total of 12 patients were consecutively enrolled in the long-term follow-up. Patients were treated with prednisone, DMARDs, or TNFBA. HBV markers, HBV DNA, and ALT levels were tested at baseline and every 2–3 months during the follow-up. This study protocol was approved by the ethics committee of our hospital, and informed consent was obtained from enrolled patients.

### Review of the literature

We search the PubMed databases using the MeSH term “hepatitis B virus” combined with the terms “DMARDs”, “steroid”, “prednisone”, “methotrexate”, “leflunomide”, “hydroxychloroquine”, “salicylazosulfapyridine”, “cyclophosphamide”, “azathioprine”, “etanercept”, “infliximab”, “adalimumab”, “rituximab” and “rheumatoid disease”. Thirty-seven articles describing 991 patients having HBV reactivation were retrieved. These patients were identified as having chronic HBV infection or past HBV infection.

### Definitions

Past HBV infection was defined as positive for anti-HBc (anti-HBc+) and negative for HBsAg (HBsAg−) [4]. Chronic HBV infection was defined as the persistent positivity of HBsAg in serum. HBV reactivation was defined as an elevation of both serum level of ALT and HBV DNA following immunosuppressive therapy.

## Results

### Baseline characteristics

From January 2008 to March 2012, a total of 12 HBV-infected patients with rheumatic diseases were consecutively enrolled in the long-term follow-up. Table 1 showed the demographic characteristic of the 12 patients. Medium age of the 12 patients was 42.5 years old (range 17–74). Seven of the 12 patients were male. Only one patient was negative for HBsAg at baseline. The level of HBV DNA was not obtained at baseline in two patients. Patients were treated with prednisone, DMARDs, and TNFBA. The medium duration of follow-up was 41 months (range 16–48). Treatment regimens were summarized in Table 1.

**Table 1:** The baseline characteristics and treatment regimen of the 12 patients enrolled

PatientNo.	Diagnosis	Sex/age	HBsAg	HBeAg	HBVDNA	Pre-emptive therapy	Therapy /follow-up months	Medications
1	DM	M/41	+	−	<10^3^	No	Pred/48	Pred 50 mg qd, now reduce to 5 mg qd
2	DM	F/74	−	−	Not done	No	Pred + AZA/16, died of cerebral infarction	Pred 50–20 mg qd + AZA 50 mg qd * 9 m
3	SLE	F/63	+	+	2.36 × 10^5^	ETV	Pred/45	Pred 40 mg qd, now reduce to 5 mg qd
4	SCLE	F/49	+	+	1 × 10^7^	ETV	Pred/30	Pred 50 mg qd, now reduce to 5 mg qd
5	AS	M/50	+	−	<10^3^	No	SASP + MTX/34	SASP 0.5 tid * 2 m MTX 10 mg qw * 24 m
6	AS	M/29	+	−	1.6 × 10^3^	No	SASP + MTX/44	SASP 1.0 bid + MTX 10 mg qw * 6 m now SASP 1.0 bid * 26 m
7	AS	M/20	+	+	2.35 × 10^7^	No	SASP/48	SASP 1.0 bid
8	RA	F/67	+	+	4.46 × 10^4^	No	MTX/47	MTX 7.5 mg qw
9	AS	M/17	+	−	<10^3^	No	SASP + MTX/47	SASP 1.0 bid + MTX 5 mg qw * 3 m now withdraw
10	AS	M/24	+	+	2.43 × 10^5^	No	ETN/46	ETN 25 mg qw * 3 m, 25 mg q2w * 2 m, 12.5 mg qm * 21 m
11	RA	F/44	+	−	<10^3^	No	HCQ/LEF + ETN/44	HCQ 0.2 bid + ETN 25 mg biw * 5 m
								LEF 10 mg qn + ETN 25 mg qw * 2 m
								ETN 25 mg qw * 20 m
12	AS	M/20	+	+	Not done	No	SASP + ETN/43	SASP 1.0 bid + ETN 25 mg biw * 3 m
								Now withdraw

*AS* ankylosing spondylitis, *AZA* azathioprine, *DM* dermatomyositis, *ETN* entanercept, *ETV* entecavir, *HBeAg* hepatitis B e antigen, *HBsAg* hepatitis B surface antigen, *HBV* hepatitis B virus, *HCQ* hydroxychloroquine, *LEF* leflunomide, *MTX* methotrexate, *Pred* prednisone, *RA* rheumatoid arthritis, *SASP* salazosulfapyridine, *SCLE* subacute cutaneous lupus erythematosus, *SLE* systemic lupus erythematosus

### Hepatitis reactivation in different treatment regimens

Prednisone was administered in four of the 12 patients (patients 1, 2, 3 and 4). Patients 3 and 4 were prescribed pre-emptive antiviral therapy. DMARDs were administered in five patients (patients 5, 6, 7, 8 and 9). The remaining three patients (patients 10, 11 and 12) were treated with the TNFBA.

**Fig. 1:**
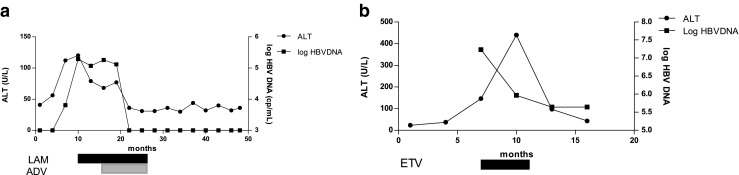
The clinical course of the two patients who developed HBV reactivation during prednisone therapy without pre-emptive therapy. **a** The clinical course of patient 1, a 41-year-old man diagnosed as DM and treated with predinisolone. **b** The clinical course of patient 2, a 74-year-old female diagnosed as DM and treated with predinisolone plus AZA. *ADV* adefovir, *AZA* azathioprine, *DM* dermatomysitotis, *ETV* entecavir, *LAM* lamivudine

**Fig. 2:**
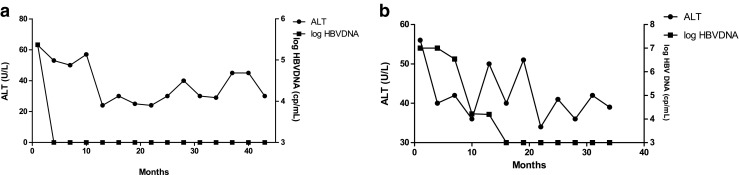
The clinical course of the two patients who received steroid therapy with pre-emptive therapy. Neither of them developed HBV reactivation. **a** Patient 3. **b** Patient 4

**Fig. 3:**
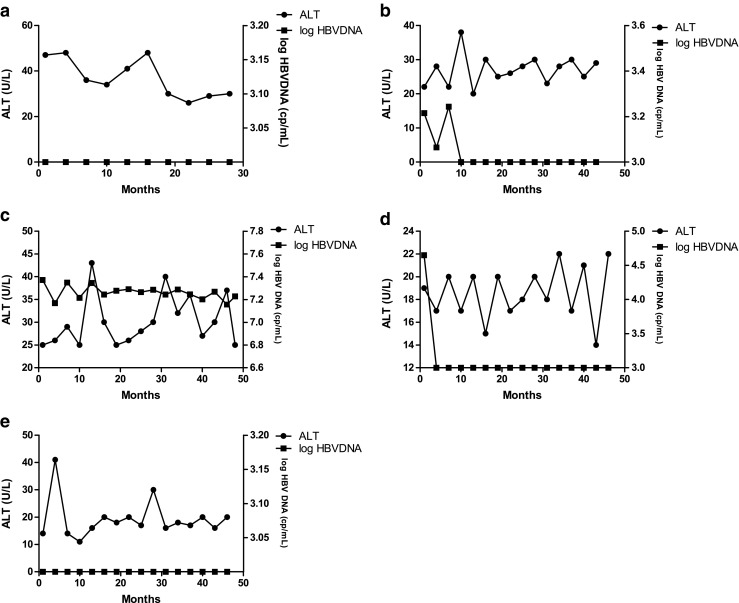
The clinical course of the five patients who received DMARDs without pre-emptive therapy. None of them developed HBV reactivation. **a** Patient 5. **b** Patient 6. **c** Patient 7. **d** Patient 8. **e** Patient 9

**Fig. 4:**
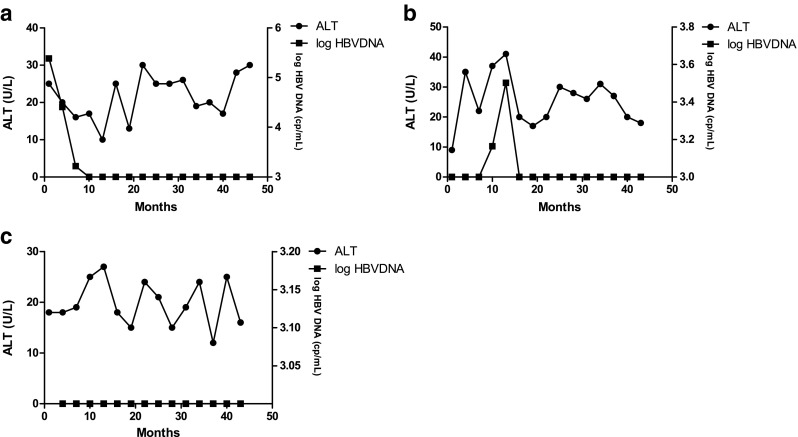
The clinical course of the three patients who underwent TNFBA without pre-emptive therapy. None of them developed HBV reactivation. **a** Patient 10. **b** Patient 11. **c** Patient 12

HBV reactivation was only documented in two patients (patients 1 and 2). Patient 1 was treated with prednisone alone, and patient 2 was treated combination therapy of prednisone and azathioprine. Neither of them received the pre-emptive therapy (Fig. 1). In contrast, the other two patients who received pre-emptive antiviral therapy before prednisone administration did not develop the HBV reactivation (Fig. 2). HBV reactivation was not observed in patients treated with DMARDs (Fig. 3) or TNFBA (Fig. 4) during the follow-up.

HBV DNA level increased in one patient after LEF administration. After the withdrawal of LEF, the level of HBV DNA turned to normal. The level of ALT remained normal during the 3-year follow-up in patients who did not develop HBV reactivation.

### Hepatitis reactivation in two cases

1. A 41-year-old man who was diagnosed with dermatomyositis in January 2008. Tests for hepatitis B virus markers, HBsAg, anti-HBc and anti-HBe yielded positive findings. The level of serum HBV DNA was under detectable limit and liver function was normal. He was treated with methylpredinisolone therapy (40 mg/day). One month later, serum level of ALT increased to 112 U/L (more than two-folds of normal limit), and serum level of HBV DNA increased to 6.41 × 10^3^ copies/ml meanwhile. Despite antiviral therapy with lamivudine, the level of ALT and HBV DNA was still high. Adefovir was added to lamivudine (10 mg/day) 4 months later. After 9 months of combination therapy, the level of ALT and HBV DNA returned to normal. LEF was added in October 2008. Serum levels of ALT and HBV DNA remained persistently normal in the following 3 years (Fig. 1a).
2. A 74-year-old woman who was diagnosed with dermatomyositis in March 2008. Regular laboratory test revealed normal serum ALT levels, and HBV serological marker was negative for HBsAg but positive for anti-HBc and anti-HBe. Serum levels of HBV DNA were undetectable before treatment with methylpredinisolone (40 mg/day) and AZA (50–100 mg/day). Six months later, no flare of ALT was observed but serological test revealed that she was positive for HBsAg. Twelve months later, hepatitis reactivation occurred. Serum levels of ALT increased to 146 U/L, and circulating HBV DNA was detectable at levels of 1.7 × 10^7^ copies/ml. Entecavir at a daily dose of 0.5 mg was started. After 3 months of antiviral therapy, serum levels of ALT decreased to 43 U/L, and serum levels of HBV DNA decreased to 1.68 × 10^3^ copies/ml (Fig. 1b). Unfortunately, the patient died of cerebral infarction during the follow-up.

### Literature review

Thirty-seven articles describing 991 patients with rheumatic diseases exposed to different immunosuppressive therapy were retrieved [5–41]. One hundred forty-four patients received steroids. Two hundred twenty-four patients received DMARDs and 620 patients received TNFBA. There were also three patients treated with anti-CD20. The summary of the literature review was shown in Table 2.

**Table 2:** The results of the literature review

Reference	Type	Patient number	HBV status (number of patients)	Medications	Pre-emptive therapy	Number of patients developing reactivation
[4, 20, 33, 38]	Case report	5	CHB (4), Past infection (1)	Prednisone	N	5
[23]	Prospective study	41	CHB (41)	Prednisone	N	21
[5]	Retrospective study	98	CHB (21), Not applied (77)	Prednisone	N	4
[8, 27]	Case report	2	CHB (1), past infection (1)	DMARDs	N	2
[12]	Prospective study	215	CHB (27), past infection (188)	DMARDs	Y (4 patients received pre-emptive therapy)	4
[13]	Prospective study	50	CHB (5), past infection (45)	DMARDs (6 patients), TNFBA (44 patients)	N	3
[31]	Retrospective study	8	CHB (8)	TNFBA	N	1
[14]	Retrospective study	49	CHB (49)	TNFBA	Y (20 patients received pre-emptive therapy)	3
[10]	Prospective study	52	CHB (14), HBV-vaccinated (19), past infection (19)	TNFBA	N	1
[11]	Prospective analysis	135	Past infection (135)	TNFBA	N	7
[32]	Retrospective study	7	CHB (7)	TNFBA	N	3
[19]	Retrospective study	92	CHB (92)	TNFBA (91 patients), DMARDs (1 patient)	N	27
[24]	Retrospective study	88	CHB (18), past infection (60)	TNFBA	Y (10 patients received pre-emptive therapy)	6
[21]	Retrospective study	60	Past infection (60)	TNFBA	N	2
[34]	Prospective study	21	Past infection (21)	TNFBA	N	0
[36]	Prospective study	67	Past infection (67)	TNFBA	N	0
[6, 7, 16, 18, 22, 25, 26, 28–30, 35, 37, 39]	Case report	17	Past infection (3), CHB (14)	TNFBA	Y (6 patients received pre-emptive therapy)	9
[15, 17, 40]	Case report	3	CHB (3)	Anti-CD30	Y (2 patients received pre-emptive therapy)	1

*CHB* chronic hepatitis b, *DMARDs* disease-modifying anti-rheumatic drugs, *HBV* hepatitis B virus, *TNFBA* tumor necrosis factor-alpha-blocking agents

With regard to HBV infection status, 609 patients had past infection and 305 patients were considered to have chronic hepatitis B. Seventy-seven patients were not detected for the serum HBV markers at baseline.

#### Patients treated with DMARDS [9, 12–14, 28]

Two hundred twenty-four patients were treated with DMARDS. Among them, 192 patients had past HBV infection and the other 32 patients were considered to have chronic hepatitis B. A total of four patients with chronic hepatitis B infection received antiviral prophylaxis therapy. HBV reactivation was reported in ten cases [9, 12–14, 28] (4.46 %) with a median follow-up of 13.2 months. MTX was administered in eight of the ten patients with HBV reactivation [9, 12–14, 28]. HBV reactivation after the initiation of LEF [13], HCQ [13], SASP [13] therapy were also reported. Antiviral therapy was started after HBV reactivation. ETV seemed to be the preferred option. A satisfied response to the antiviral therapy was observed in most cases. Two patients [13] did not receive antiviral therapy despite the HBV reactivation. Serum levels of HBV DNA were markedly elevated in one case but remained persistently normal in the other case. Patient with bad outcome was only reported by Satoshi Ito [28], in which the patient died of fulminant hepatitis after a 28-month follow-up in spite of the treatment with interferon-b.

#### Patients treated with TNFBA [7, 8, 10–12, 14, 15, 17, 19, 20, 22, 23, 25–27, 29–33, 35–38, 40]

Six hundred twenty patients were treated with TNFBA. Among them, 416 patients had past infection with HBV and 204 patients had chronic HBV infection. Antiviral prophylaxis therapy was administered in 36 patients with chronic viral infection. HBV reactivation was reported in 59 (9.52 %) patients, in whom 13 patients were with past infection while the remaining 46 patients were chronic infection. Treatment with ETA or INF for rheumatic diseases was reported in most cases. Followed by the appearance of viral reactivation, the antiviral therapy was immediately started. Bad outcome was seen in only one case [23], in which the patient suffered liver failure and died after 26 months of follow-up.

#### Patients treated with steroids [5, 6, 21, 24, 34, 39]

Steroids were usually co-administered with DMARDs or TNFBA for treating rheumatic diseases. This may explain why only 6 articles reporting 144 patients were included. However, the effect of steroids on hepatitis reactivation cannot be denied. A total of 30 (one with past infection and the others with chronic infection) of the 144 patients experienced the HBV reactivation after a median time of 9.8 months. Serum HBV markers prior to steroid therapy were detailed in 67 patients. Among them, only one patient was defined as past infection and the other 66 patients were chronic infection. Four out of the seven articles were case reports [5, 21, 34, 39] concerning six patients with rheumatic diseases affected by hepatitis B virus. Bae [39] reported a fatal case of hepatitis B virus (HBV) reactivation during long-term, very-low-dose steroid treatment in an inactive HBV carrier. The patients was treated with prednisone combined with DMARDs (SSZ and HCQ), however the reactivation of HBV was mainly attributed to the steroid according to the author's opinion. There are two cases [21, 34] that reported steroid monotherapy for treating rheumatoid disease. None of the patients received prophylaxis treatment and developed HBV reactivation after 5 and 9 months of the administration, respectively. Another case [5] showed hepatic failure due to fibrosing cholestatic hepatitis in a patient with pre-surface mutant hepatitis B virus and mixed connective tissue disease treated with prednisolone and chloroquine. Similar to the patient treated with low dose of steroid [39], chloroquine was prescribed to supplement the steroid treatment. Both patients died of liver failure. HBV reactivation in patient with past HBV infection after steroid therapy [34] was also reported. A brief report of prospective study on a comparison of a standard-dose prednisone regimen and mycophenolate mofetil combined with a lower prednisone dose in Chinese adults with idiopathic nephrotic syndrome who were carriers of hepatitis B surface antigen [24] were included in this study. The result of study showed that rate of reactivation was higher in prednisone regimen than in combination therapy group, confirming that the impact of prednisone on the viral reactivation. Another retrospective study [6] reported HBV reactivation following GC therapy for a case series of pemphigus vulgaris and dermatomyositis. Four out of the 98 patients finally had a reactivation after a median time of 10.5 months.

#### Patients treated with anti-CD20 [16, 18, 41]

Rituximab is the representative of anti-CD20, which is also a kind of biological DMARD. Only one patient [18] underwent the HBV reactivation and was treated with LAM combined with tenofovir. A good response to the treatment was received.

## Discussion

Clinical cases about HBV reactivation in patients with rheumatic disease after immunosuppressive therapy have been increasing, which suggests the possibility of HBV reactivation in the patients receiving immunosuppressive treatment for rheumatic diseases. Recent researches of HBV reactivation leading to serious complications have been described in patients with rheumatic disease undergoing treatment with biological agents, mainly including infliximab and rituximab [19, 42]. Considering the potential risk of biological agents, pre-emptive antiviral treatment is recommended in HBsAg-positive patients at the commencement of TNFBA treatment [8].

We presented the result of three patients who received biological agents (entanercept) in this study. HBV reactivation occurred in none of them. There are, however, observations from our literature review suggesting that 59 patients in 620 cases finally experienced the hepatitis reactivation. The result suggests the potential of TNFBA to induce viral reactivation. Once the anti-TNF agents were withdrawn and antiviral therapy was given, the level of liver enzyme and HBV DNA decreased to normal [43] and patients came through in most cases. Hepatitis reactivation usually occurred 30–60 days after immunosuppressive therapy. Antiviral prophylaxis treatment is recommended in HBsAg-positive patients. Since the publication and selection bias cannot be avoided, a bigger and longer follow-up study is required to clarify the influence on hepatitis reactivation after anti-TNF-alpha treatment.

SASP and MTX are the commonly single-use DMARDs in rheumatic patients. The possibility of DMARDs to induce HBV reactivation is still a controversial issue in recent studies. In our serial cases, five patients received SASP therapy, and none of them developed hepatitis reactivation regardless of serum levels of HBV DNA prior to SASP therapy. No research approves that SASP induce hepatitis reactivation. We believe SASP is safe on hepatitis B patients. Four patients were treated with MTX therapy and no HBV reactivation was observed during follow-up. In our literature review, most cases were treated with MTX as a preferred choice. A few researches reported HBV reactivation during or after MTX therapy [17, 28, 44]. One of them progressed to fatal hepatic failure in the end [44]. According to the result of the literature review, DMARDs seem to be the safest medicine considering only 4.46 % of the patients experienced HBV reactivation after the treatment.

The efficacy of prednisone on hepatitis reactivation is proved again. In this study, HBV reactivation occurred in patients who did not receive pre-emptive therapy. Results from some research suggested that the risk of hepatitis reactivation depends on the dosage of prednisone [24]. However, in our literature review, this assumption was not proved. Prophylaxis antiviral therapy is necessary in those hepatitis B-infected patients who will receive prednisone therapy. Optimal agent for pre-emptive therapy is still controversial. The benefits of LAM may be limited due to resistance and entecavir is recommended as first choice for pre-emptive therapy. HBV reactivation induced by prednisone has been investigated thoroughly in cancer patients receiving chemotherapy treatment and some consensus has been made. However, in the field of rheumatism, the subject seemed to be anything but a hotspot. Thirty of the 144 patients experienced HBV reactivation in the literature review, which strongly suggested the necessity to prescribe antiviral pre-emptive therapy.

HBV reactivation also occurs in HBV-infected patients who were negative for HBsAg but positive for anti-HBc. In this study, a 74-year-old woman who was negative for HBsAg before the commencement of prednisone therapy experienced HBV reactivation 11 months after prednisone administration. Reactivation of HBV infection has been reported in HBsAg-negative patients who received chemotherapy [45] for lymphoma. Observation of the literature review also confirmed the possibility of viral reactivation in patients with past HBV infection treated with immunosuppressive therapy for rheumatic diseases. Nineteen of the 609 patients (3.20 %) with past infection experienced reactivation after immunosuppressive therapy. Taking these lines of evidence together, serum levels of ALT and HBV DNA should be monitored not only in patients who were chronically infected, but also in patients who have past infection (negative for HBsAg and positive for anti-HBc).

The time of HBV reactivation is still not confirmed, based on our study. Not surprisingly, reactivation was observed during the treatment with immunosuppressive therapy. However, even after the drug withdrawal, the reactivation still occurred [8]. Therefore, the whole course of the therapy, instead of the first few months, ought to be monitored closely.

In conclusion, HBV reactivation also occurs in patients with rheumatic diseases treated with immunosuppressive therapy. Pre-emptive therapy is proved beneficial in cancer patients with hepatitis B. However, pre-emptive therapy in patients with rheumatic diseases treated with different immunosuppressive therapy is still a matter of controversy. In terms of our study, the benefits of pre-emptive therapy definitely exceed the harm of it in patients receiving steroids. Entecavir is recommended as the optimal agent against HBV reactivation. On the other hand, DMARDs is shown to be quite safe in rheumatic patients with HBV infection so that pre-emptive therapy may not be recommended in these patients. TNFBA is considered safe in HBsAg-negative patients, while pre-emptive is required in patients who are positive for HBsAg. Unnecessary antiviral therapy may induce virus mutation and further increases the economic burden. Despite the lower risk of HBV reactivation in patients with rheumatic diseases who are candidates for DMARDs and TNFNA therapy, such patients should be followed-up periodically and tested for ALT and HBV DNA.

## Abbreviations

ADV: Adefovir
ALT: Alanine aminotransferase
AS: Ankylosing spondylitis
AZA: Azathioprine
CHB: Chronic hepatitis B
DM: Dermatomyositis
DMARDs: Disease-modifying anti-rheumatic drugs
ETN: Entanercept
ETV: Entecavir
HBeAg: Hepatitis B e antigen
HBsAg: Hepatitis B surface antigen
HBV: Hepatitis B virus
HCQ: hydroxychloroquine
LAM: Lamivudine
LEF: Leflunomide
MTX: Methotrexate
Pred: Prednisone
RA: Rheumatoid arthritis
SASP: Salazosulfapyridine
SCLE: Subacute cutaneous lupus erythematosus
SLE: Systemic lupus erythematosus
TNFBA: Tumor necrosis factor-alpha-blocking agents
